# Understanding the Spatial Predictors of Malnutrition Among 0–2 Years Children in India Using Path Analysis

**DOI:** 10.3389/fpubh.2021.667502

**Published:** 2021-07-30

**Authors:** Monika Singh, Md Sayeef Alam, Piyusha Majumdar, Bhaskar Tiwary, Hina Narzari, Yodi Mahendradhata

**Affiliations:** ^1^District Resource Unit, Bihar Technical Support Program, Care India, West Champaran, India; ^2^Institute of Health Management Research, IIHMR University, Jaipur, India; ^3^Department of Fertility Studies, International Institute for Population Sciences, Mumbai, India; ^4^Concurrent Measurement and Learning Unit, Bihar Technical Support Program, Care India, Saharsa, India; ^5^Department of Public Health and Mortality Studies, International Institute for Population Sciences, Mumbai, India; ^6^Department of Health Policy and Management, Faculty of Medicine, Public Health and Nursing, Universitas Gadjah Mada, Yogyakarta, Indonesia

**Keywords:** wasting, underweight, path analysis, spatial analysis, stunting

## Abstract

**Background:** Despite several programs and policies to turn down the burden of malnutrition in the country, the rank of India in the Global Hunger Index (GHI) is 102 among 117 countries, which indicates a serious hunger situation. It is essential to design more specific interventions by focusing on the key determinants that may directly or indirectly influence malnutrition in India.

**Methods:** Utilizing data from the National Family and Health Survey-4 (NFHS) (2015-16), we developed a structural equation model to find the direct, indirect, and total effect of various determinants on stunting, wasting, and underweight. We used spatial analysis to identify local occurrences of factors that are critical in controlling malnutrition. A *p*-value of 0.05 was considered to be significant throughout the study. Analysis was performed using STATA (version 15.1MP) and GeoDa software (version 1.14).

**Results:** A final sample of 90, 842 children of 0–24 months of age was selected for the analysis. The CFI and TLI values of 0.98 and 0.93, respectively, are indicative of a good fit model. Moran's I value of global spatial autocorrelation for the widespread presence of diarrhea, poor drinking water source, exclusive breastfeeding, low birth weight, no prenatal visits, poor toilet facility was observed to be 0.446, 0.638, 0.345, 0.439, 0.620, and 0.727, respectively.

**Conclusion:** A robust direct relation was observed for diarrhea, exclusive breastfeeding, and children born with stunting, underweight, and wasting. The variables associated indirectly with the outcome variables were the education of the mother, residence, and desired pregnancy. The identification of hotspots through spatial analysis would help revive control strategies in the affected area according to geographical needs. It is extensively addressed that interventions related to health and nutrition during the first 1, 000 days of life is crucial to seize the upshoot of growth floundering among children.

## 1. Introduction

A powerful window of fortuity is considered from the day a woman conceives till the second birthday of the child. The world would be free from the burden of malnutrition if we invest in the well-being of women and children in this period. Malnutrition remains to be one of the significant cause of morbidity & mortality among under-five aged children, particularly in low- and middle-income countries (LMICs) (1, 2). The WHO reported that 47 million children under 5 years of age are wasted, and 144 million are stunted. As per the recent national-level representative study, in India, 38.4, 21, and 35.7% of children under 5 years of age suffer from stunting, wasting, and underweight.

The problem is of particular essence for vulnerable infants and young children because they require energy and nutrient-dense foods to grow and develop physically and mentally (3). Further, increased rates of malnutrition imperil future economic growth by diminishing psychological and physical capabilities of the entire population (4). It leads to high levels of chronic illness and disability in adult life (5). In the developing world, shortness of diversity in food and nutrition is a critical problem in poor populations. Because of these reasons, specific recommendations are included for dietary diversity in the recently updated guideline for the complementary feeding of breastfed child aged from 6 to 23 months (6, 7).

The nutritional status of mother is a vital determinant of nutritional status of a child. Hence, should be considered in a program designed to improve the health of a child (8). Birth weight has a vigorous negative effect on subsequent weight gain, which becomes more when birth weight is considered as an endogenous variable (9). More diverse diets are seen in families with high incomes and resources. In addition, they are likely to have better access to health care and better environmental conditions (10). Many reasons contribute to the better physical and mental growth of children in wealthier households; an improved nutritional state may be an significant way to translate household wealth and resources into better outcomes for children (11).

The rank of India in the Global Hunger Index (GHI) is 102 among 117 countries, which indicates a serious hunger situation in the country (12). Malnutrition explains around 69% of deaths in children below 5 years of age in the year 2019 (13). Minimizing the burden of malnutrition in children is one of the major concerns of government and non-government organizations' working toward improving health of the child. Child malnutrition is a public health priority. One-third of deaths can be attributed, particularly, when not invested from the day women conceive until the second birthday of the child (14). Therefore, we first aimed to explore the direct, indirect, and total effects of socio-demographic, household, environmental factors on stunting, wasting, and underweight among 0–2 years of children. It has not been reported from India to date. Second, the study also reports the spatial evaluation of low birth weight, diarrhea, toilet facility, prenatal visits and breastfeeding, as these are important predictors of malnutrition (15, 16). Comprehensive facts found geographically through spatial analysis would help the government in the arbitration of health policy more effectively (17). Constituting discrete interventions by emphasizing the causal factors of malnutrition is essential to fight against it.

## 2. Materials and Methods

### 2.1. Data Set

A nationally representative survey, the National Family Health Survey-4 (NFHS), was used to conduct the presented study. Data are available from 28 states to 8 union territories (UTs) of India. The applied sampling structure (a two-stage stratified sampling frame) was based on the census of India from 2011. The primary stage units being the villages and the census enumeration blocks for rural and urban areas, respectively. In the next stage, i.e., the second stage, from every preceding sampling unit/village/block, household were picked out for the survey grounded on the probability of systematic sampling. It was a survey steered by various regional organizations, which were harmonized by International Institute for Population Sciences (IIPS), Mumbai.

### 2.2. Variables

The National Family Health Survey provided data on 249,809 children of age group 0–59 months. For the study, a final sample of 90,842 children of 0–24 months of age was selected for further analysis. The outcome variables were stunting, wasting, and underweight. Other exogenous variables included for the study consisted of birth weight (low, normal, and high); exclusive breastfeeding (yes, no); children ever born (single child, two children, three children, four children, five children, and more than five children) and prenatal visits (yes or no). The endogenous variables included in the model were diarrhea (no; yes, in last 2 months); body mass index (low, normal, and high); residential area (rural or urban); household wealth index (poor, middle income, and rich); mother's education (illiterate, 6–8, 9–10, 11–12, and more than 12 years), wanted pregnancy when became pregnant (then, later, and no more). For exclusive breastfeeding of 21,151 children, last-born children of 0–5 months were considered as per Demographic and Health Survey (DHS) guidelines.

### 2.3. Statistical Analysis

#### 2.3.1. Path Analysis

**Figure 1:**
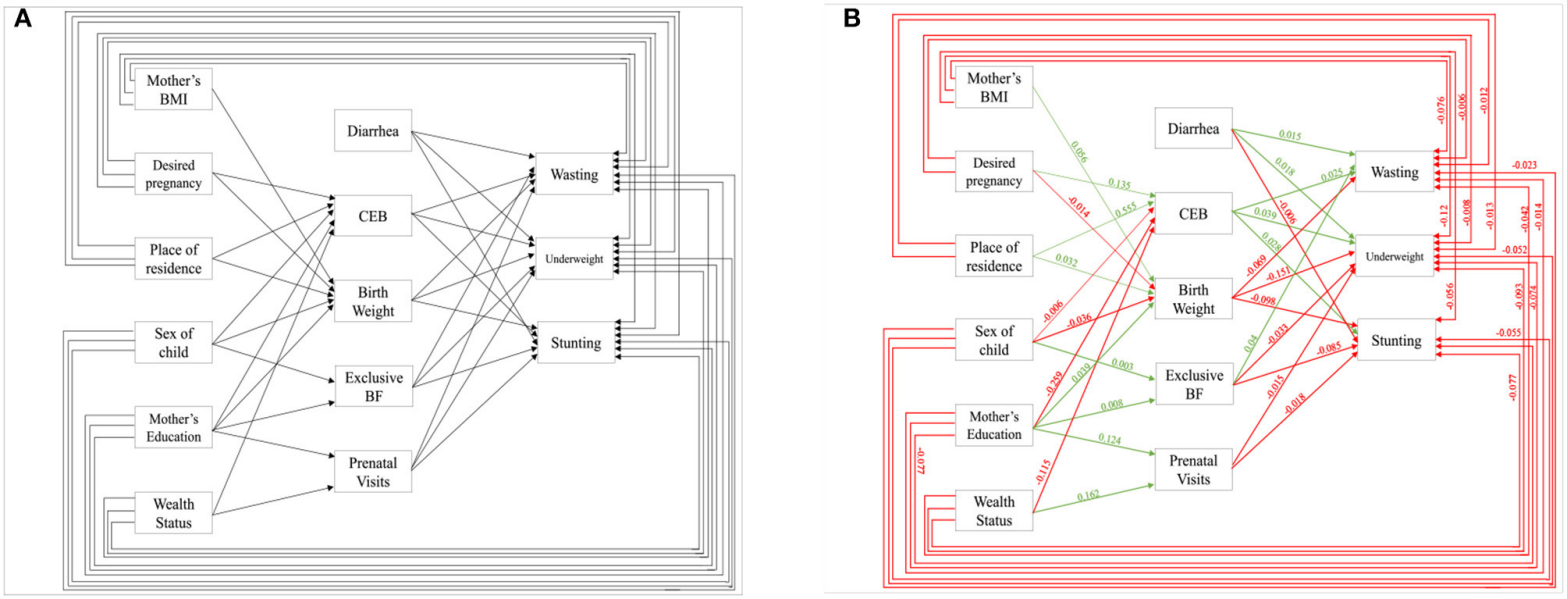
Pathways toward malnutrition, **(A)** hypothesized model, **(B)** final model with significant effects (Source: Author's Created).

A path analysis was performed to quantify the hypothetical pathway (Figure 1) *via* fitting a series of regression equations if the model remains unaffected by confounding factors (18). To estimate the parameters of the model, we used weighted least square adjusted for mean and variance (19). Four model fit indices, each associated with a specific feature of the model, were taken into consideration to quantify the degree of correspondence between the data and the hypothesized models (19, 20). According to Hu and Bentler (21) and Kenny and McCoach (22) the combined criteria for the goodness of fit including or Comparative Fit Index (CFI) and Root Mean Square Error of Approximation (RMSEA), or Standardized Root Mean Square Residual (SRMR) together helps to decide on accepting a model that has been considered. The CFI and Tucker-Lewis Index (TLI) are both incremental fit indices, and values > 0.95 for both these indices specify a very good fit of the model (23). The RMSEA values are considered indicative of close fit if they are ≤0.05, and values up to 0.08 are considered acceptable (24). In addition, the CFI and TLI values equal to or above 0.90 are considered proof of acceptable fit. The complete independent effect on the exogenous variable by an endogenous variable is the direct effect. In contrast, the effect of endogenous variables on exogenous variables *via* some other endogenous variables is known to be indirect effects. The total effect is the summation of direct and indirect effects on exogenous variables *via* the endogenous one. At five levels it was considered to be statistically significant, and the analysis was run by using STATA (version 15.1 MP).

- Chi-square test for model fit (χ^2^)
- Comparative Fit Index (CFI)
- Tucker-Lewis Index (TLI)
- Root Mean Square Error of Approximation (RMSEA)

#### 2.3.2. Investigational Spatial Analysis

Geographical locations with the widespread occurrence of low birth weight, diarrhea, toilet facility, prenatal visits, and breastfeeding were obtained through spatial cluster detection. Moran's I value of global spatial autocorrelation was computed to forecast the robustness, pattern, and overall clustering of data. District measures of variables were recognized using LISA statistics (25). LISA was exerted to estimate supremacy of individual districts' on the amplitude of global statistics and to locate the clusters (26). A significance map presents the territory with a statistically significant LISA statistic value (27). Geographical locations with widespread occurrence are called the hotspots and are encompassed by various other geographical units with high prevalence.

Similarly, a cold spot is a geographical location with low prevalence encompassed by various other geographical units with low prevalence (28). To provide weights, Queens's first-order contiguity matrix was deployed (29). A *p*-value of 0.05 was considered significant throughout. The analysis was executed through GeoDa software (version 1.14).

## 3. Results

### 3.1. Path Analysis

We hypothesized a model in Figure 1A based on works of literature available. Using path analysis, we evaluated the effects of various variables through structural equation modeling (SEM) as shown in Figure 1B on stunting, wasting, and underweight controlling the confounders. The green line indicates a significant positive effect, while the red line indicates a significant negative effect. The estimates of path analysis are presented in Table 1.

**Table 1:** Estimates of hypothesized pathway effect on stunting, wasting, and underweight among children of 0–2 years of age.

**Effect**	**Predictor variables**	**Stunting**		**Wasting**		**Underweight**	
		**Estimate (SE)**	***p*** **-value**	**Estimate (SE)**	***p*** **-value**	**Estimate (SE)**	***p*** **-value**
	Prenatal-Visit	0.001 (0.0004)	<0.001	–0.001 (0.0002)	<0.001	0.001 (0.0002)	<0.001
	Mother's BMI	–0.004 (0.0003)	<0.001	–0.003 (0.0002)	<0.001	–0.007 (0.0005)	<0.001
	Sex of child	0.003 (0.0004)	<0.001	0.002 (0.0003)	<0.001	0.005 (0.0005)	<0.001
Indirect	Place of residence	0.001 (0.0003)	<0.001	0.001 (0.0002)	<0.001	0.002 (0.0002)	<0.001
	Household wealth index	–0.003 (0.0004)	<0.001	–0.002 (0.0004)	<0.001	–0.004 (0.0004)	<0.001
	Mother's education	–0.005 (0.0005)	<0.001	–0.003 (0.0004)	<0.001	–0.006 (0.0005)	<0.001
	Desired pregnancy	0.006 (0.0008)	<0.001	0.004 (0.0007)	<0.001	0.008 (0.0009)	<0.001
	Prenatal-visit	–0.02 (0.004)	<0.001	0 (0.004)	0.847	–0.02 (0.004)	<0.001
	Mother's BMI	–0.04 (0.003)	<0.001	–0.06 (0.003)	<0.001	–0.09 (0.003)	<0.001
	Sex of child	–0.05 (0.003)	<0.001	–0.02 (0.003)	<0.001	–0.05 (0.003)	<0.001
Direct	Place of residence	0 (0)	0.462	0 (0)	0.006	0 (0)	0.002
	Household wealth index	–0.04 (0.002)	<0.001	–0.02 (0.002)	<0.001	–0.05 (0.002)	<0.001
	Mother's education	–0.03 (0.001)	<0.001	0 (0.001)	0.001	–0.02 (0.001)	<0.001
	Desired pregnancy	–0.01 (0.004)	0.135	–0.01 (0.003)	0.075	–0.01(0.004)	0.015
	Prenatal-visit	–0.02 (0.004)	<0.001	0 (0.004)	0.726	–0.02 (0.004)	<0.001
	Mother's BMI	–0.05 (0.003)	<0.001	–0.06 (0.003)	<0.001	–0.1 (0.003	<0.001
	Sex of Child	–0.05 (0.003)	<0.001	–0.02 (0.003)	<0.001	–0.04 (0.003)	<0.001
Total	Place of residence	0 (0)	<0.001	0 (0)	0.962	0 (0)	0.266
	Household wealth index	–0.04 (0.002)	<0.001	–0.02 (0.002)	<0.001	–0.05 (0.002)	<0.001
	Mother's education	–0.03 (0.001)	<0.001	–0.01 (0.001)	<0.001	–0.03 (0.001)	<0.001
	Desired pregnancy	0 (0.004)	0.956	0 (0.003)	0.623	0 (0.004)	0.828

*SE, Standard Error; P-Value, Significant at 0.05*.

#### 3.1.1. Indirect Effect

The variables of the model proved to be a significant contributor through indirect effect toward stunting, wasting, and underweight. Prenatal visits, education, and BMI of the mother, and household wealth index were found to negatively affect wasting. In contrast, the sex of the child, residence, and desired pregnancy had a significant positive effect on wasting. Similarly, for underweight and stunting prenatal visits, sex of the child, place of residence, and desired pregnancy had a significant positive indirect effect, whereas BMI of the mother, household index, and education of the mother had a significant negative indirect effect.

#### 3.1.2. Direct Effect

For wasting, exclusive breastfeeding, children ever born and diarrhea had a significant direct positive effect. In contrast, birth weight of a child, sex of the child, BMI of the mother, wealth index, and desired pregnancy had a significant negative direct effect on prenatal visits. Desired pregnancy had insignificant direct effects on stunting.

For birth weight of children born underweight, exclusive breastfeeding, prenatal visits, BMI of the mother, sex of the child, household index, education of the mother, and desired pregnancy had a significant negative direct effect. In contrast, children are ever born and diarrhea had a significant direct positive effect. For stunting, only children ever born had a significant positive direct effect, while birth weight of the child's, exclusive breastfeeding, prenatal visits, BMI of the mother, sex of child, household wealth index, and education of mother's had a significant negative effect, whereas diarrhea, place of residence, and desired pregnancy had direct effects, which were not significant at all.

#### 3.1.3. Total Effect

For stunting, children ever born had a significant positive total effect. In contrast, birth weight of children's, exclusive breastfeeding, prenatal visits, BMI of the mother, sex of child, household wealth index, and education of the mother had a significant total negative effect and diarrhea and desired pregnancy had non-significant total effects. For wasting exclusive breastfeeding, children ever born, diarrhea had a significant positive total effect. In contrast, birth weight of the child, education, and BMI of the mother, sex of child, and household wealth index had a significant total negative effect, prenatal visits, place of residence, and desired pregnancy had non-significant total effects. For underweight, children ever born and diarrhea had a significant positive total effect, whereas birth weight of a child, exclusive breastfeeding, prenatal visits, sex of the child, education of the mother and wealth index, and BMI of the mother had significant total negative effects and place of residence and desired pregnancy had non-significant total effects.

#### 3.1.4. Goodness of Fit

The CFI and TLI values equal to or above 0.90 are considered proof of acceptable fit. The model fit indices of this study, i.e., CFI, TLI, and RMSEA values are 0.980, 0.927, and 0.036, respectively. The p close test was conducted to determine if the model departed significantly from the model which was a close fit (i.e., with RSMEA ≤ 0.05). In this study, we got a p-close test value of 1, which is not significant, and hence, it can be concluded that the model presented is a very good fit.

### 3.2. Spatial Analysis

#### 3.2.1. Overall Clustering of Data

**Figure 2:**
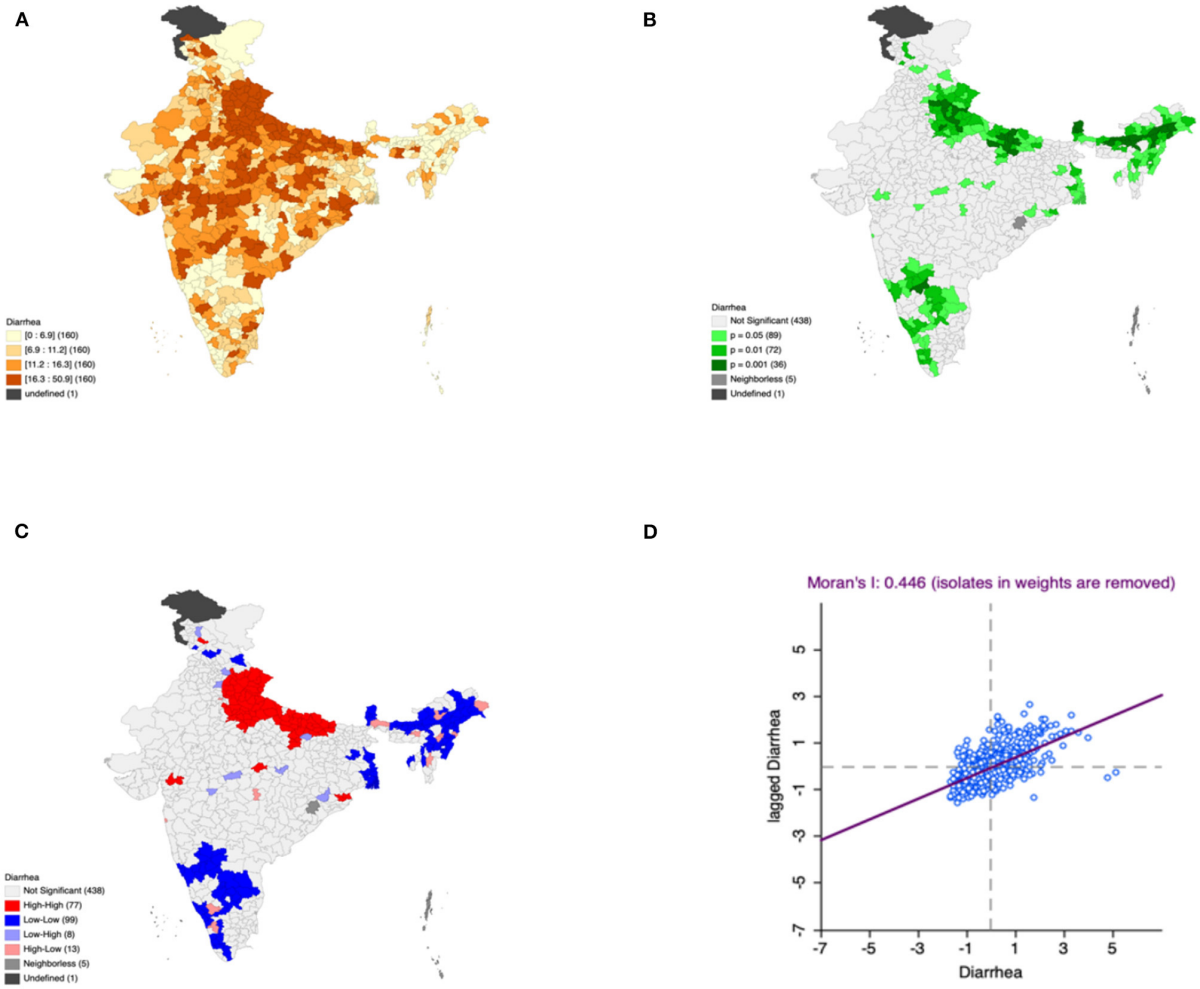
Presence of diarrhea **(A)** significance map, **(B)** LISA significance map, **(C)** LISA cluster map, **(D)** moran scatter plot for the year 2015–2016 in India (Source: Author's Created).

**Figure 3:**
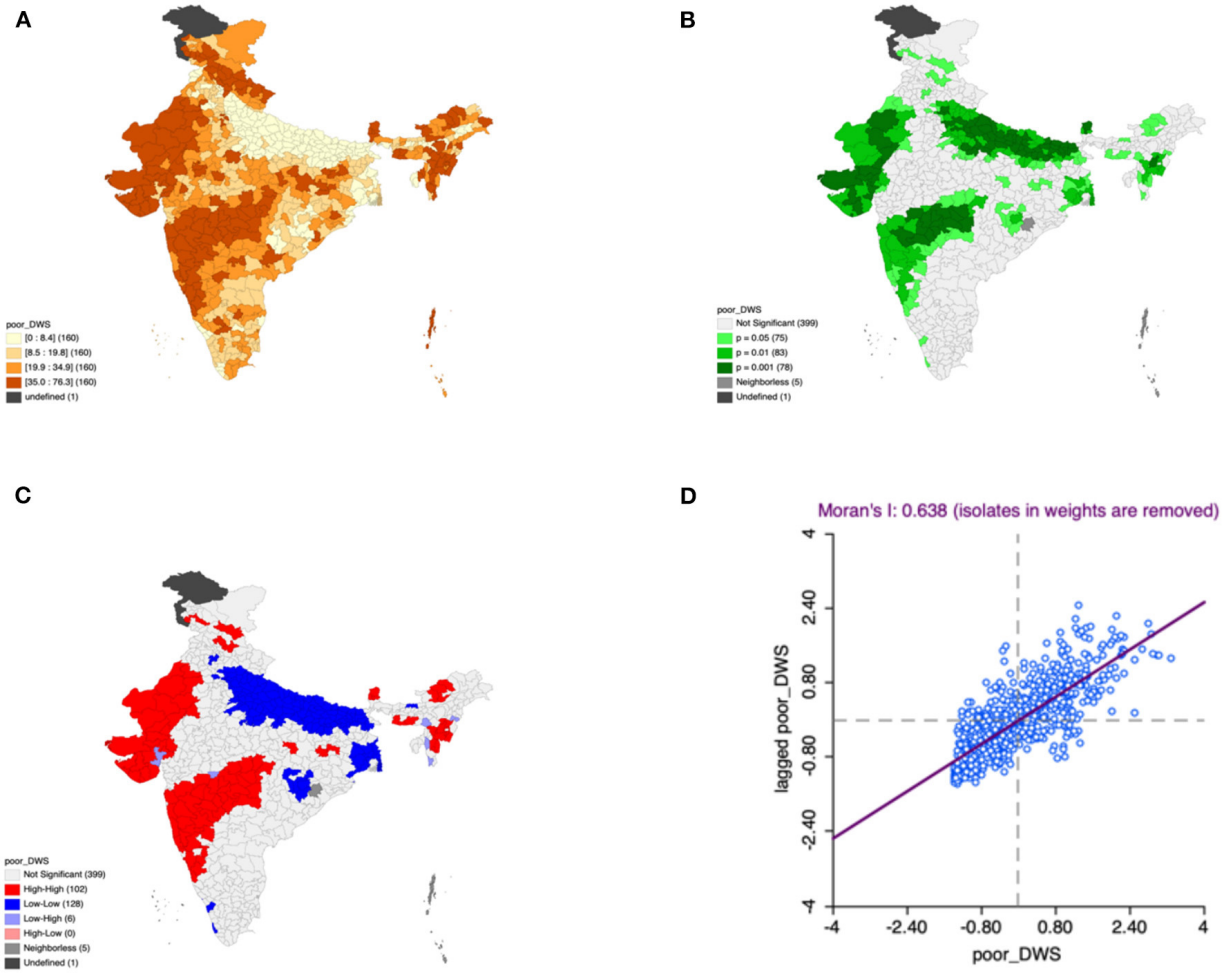
Presence of poor drinking water **(A)** significance map, **(B)** LISA significance map, **(C)** LISA cluster map, **(D)** Moran scatter plot for the year 2015–2016 in India (Source: Author's Created).

**Figure 4:**
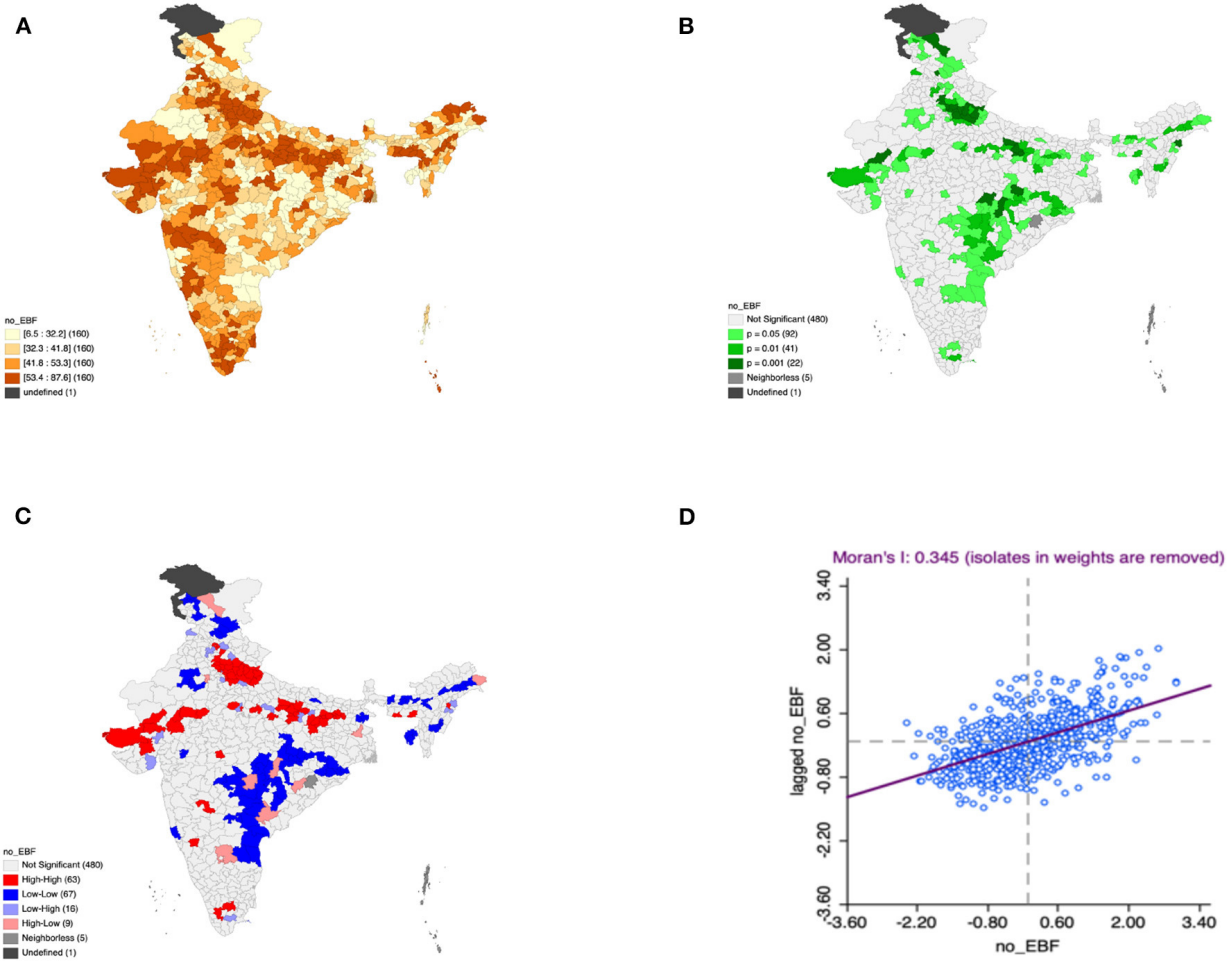
Presence of no exclusive breastfeeding **(A)** significance map, **(B)** LISA significance map, **(C)** LISA cluster map, **(D)** Moran scatter plot for the year 2015–2016 in India (Source: Author's Created).

**Figure 5:**
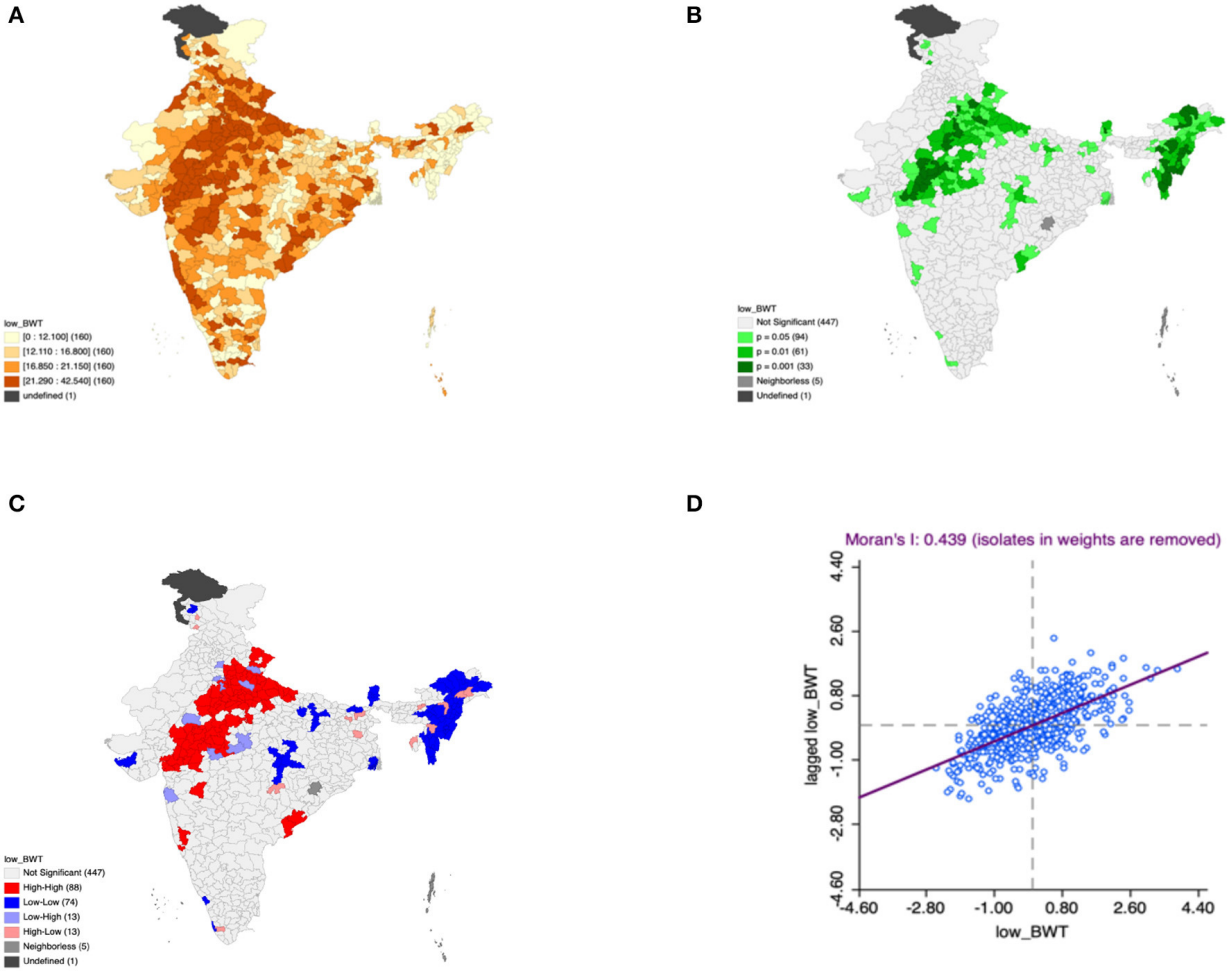
Presence of low birth weight **(A)** significance map, **(B)** LISA significance map, **(C)** LISA cluster map, **(D)** Moran scatter plot for the year 2015–2016 in India (Source: Author's Created).

**Figure 6:**
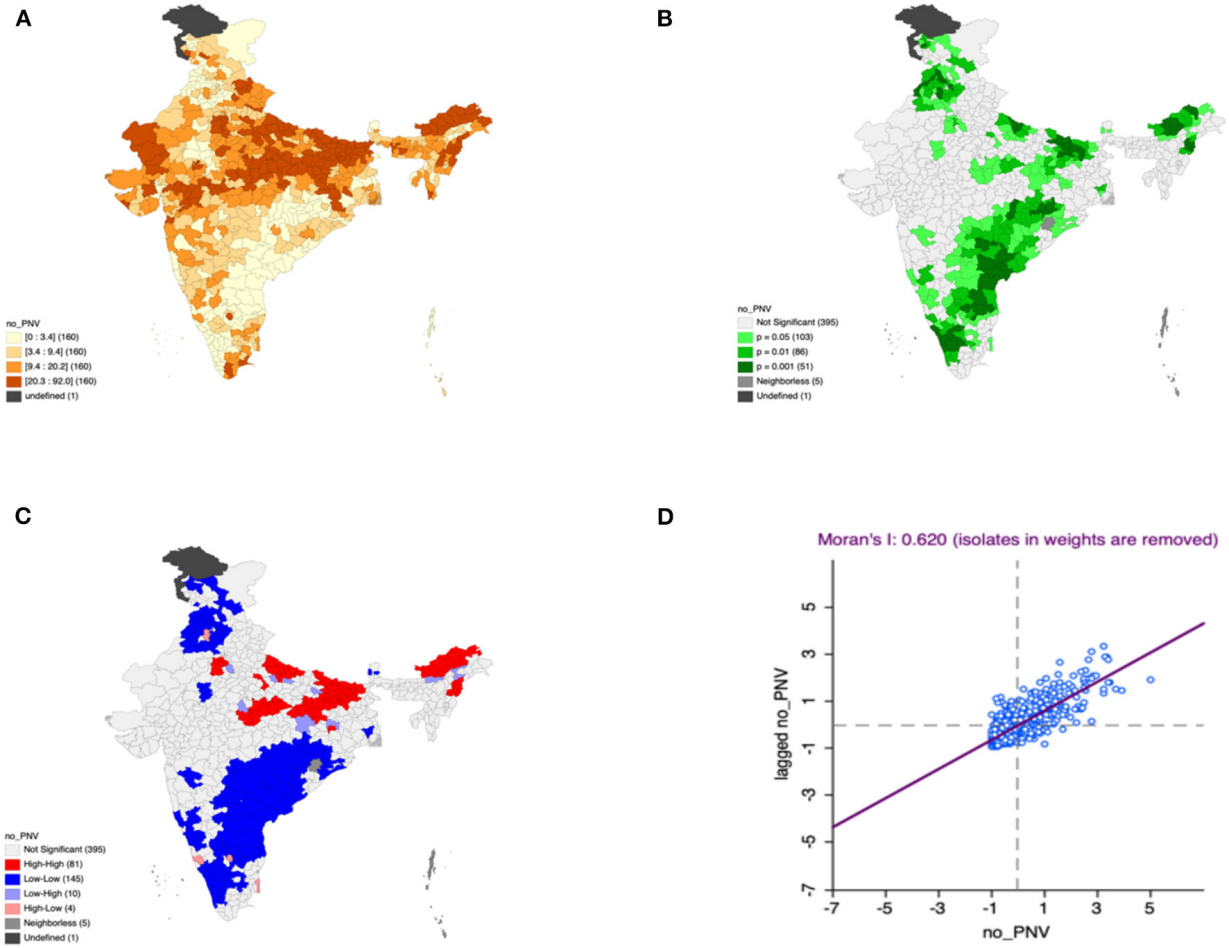
Presence of no pre-natal visits **(A)** significance map, **(B)** LISA significance map, **(C)** LISA cluster map, **(D)** Moran scatter plot for the year 2015–2016 in India (Source: Author's Created).

**Figure 7:**
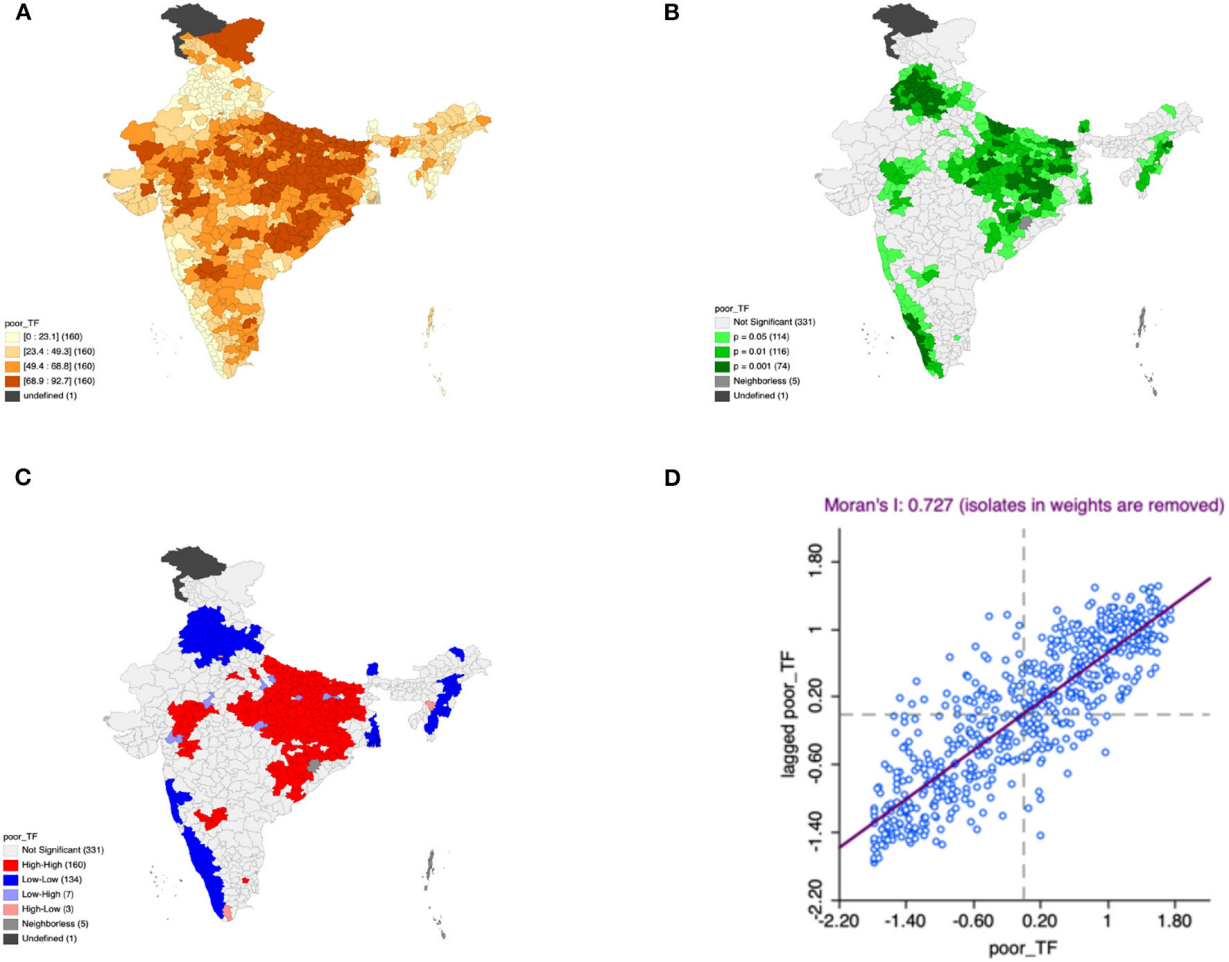
Presence of poor toilet facility **(A)** significance map, **(B)** LISA significance map, **(C)** LISA cluster map, **(D)** Moran scatter plot for the year 2015–2016 in India (Source: Author's Created).

Moron's scatter plot in Figures 2D–7D renders a visual portrayal of data assigned to the shapefile and the spatial associations about each district–level reflection. Global spatial autocorrelation for the prevalence of diarrhea, drinking water source, exclusive breastfeeding, low birth weight, prenatal visits, toilet facility is observed to decrease significantly over time from 0.446, 0.638, 0.345, 0.439, 0.620, and 0.727, respectively. The result records seclusion of the significant cluster elements, which is an intimation to include a strand of geography in the model for ascertaining the determinants of malnutrition (Figures 2A–7A).

#### 3.2.2. Detection of Hotspots

This study revealed 77 hotspots for diarrhea, 102 hotspots for a poor drinking water source, 63 hotspots for exclusive breastfeeding, 88 hotspots for low birth weight, 81 hotspots for prenatal visits, and 160 hotspots for poor toilet facility in the region studied. The hotspots and cold spots for various variables are presented in Figures 2C–7C. The significance map portrayed locale with significant local Moran statistics as depicted in Figures 2B–7B. The observed gray locations on the map of India are because of a lack of data in the NFHS 2015−16. Table 2 describes top five districts in India with extreme percentages of factors that affect malnutrition as evidenced from NFHS-4 data.

**Table 2:** Describing top 5 districts with extreme percentage of factors affecting malnutrition among children aged 0–2 years (NFHS -4, 2015–16).

**Characteristics**	**Lowest percentage district, state**	**Percentage screened**	**Highest percentage- district, state**	**Percentage screened**
	Rampur, UP	87.56	Kabeerdham, Chhattisgarh	14.77
	Muzaffarnagar, UP	87.37	Bilaspur CT, Chhattisgarh	14.35
Less exclusive breastfeeding	Meerut, UP	82.41	Jhunjhunu, Rajasthan	13.93
	Ambedkar Nagar, UP	81.88	Narsimhapur, MP	13.87
	Jaunpur, UP	78.64	Rajnandgaon, Chhattisgarh	13.37
	Latehar, Jharkhand	92.67	Palakkad, Kerala	0
	Simdega, Jharkhand	92.49	Ludhiana, Punjab	0
Poor toilet facility	Siddhi, MP	92.28	Lakshadweep	0
	Madhepura, Bihar	90.51	West DL, NCT of Delhi	0
	Balangir, Odisha	90.39	Mahe, Puducherry	0
	Ganganagar, Rajasthan	76.32	Gonda, UP	0
	Rajkot, Gujarat	71.71	Purnia, Bihar	0
Poor water source	Buldana, Maharashtra	71.07	Siddharth Nagar, UP	0
	Mahesana, Gujarat	69.64	Sant Kabir Nagar, UP	0
	West SK, Sikkim	69.18	Bhojpur, Bihar	0
	East Kameng, AP	92.05	Ramanagara, Karnataka	0
	Mon, Nagaland	75.9	Kozhikode, Kerala	0
No prenatal-visit	Longleng, Nagaland	71.88	Bargarh, Odisha	0
	Bahraich, UP	68.72	Ernakulam, Kerala	0
	Balarampur, UP	67.81	Kollam, Kerala	0
	Ramban, J & K	50.87	Wayanad, Kerala	1.1
	Kishtwar, J & K	48.18	Tirap, AP	0.83
Diarrhea-yes	Siddharth Nagar, UP	42.18	Sonitpur, Assam	0.82
	Mau, UP	39.35	Karbi Anglong, Assam	0.81
	Meerut, UP	36.99	Darrang, Assam	0.78
	Mandsaur, MP	42.54	West Siang, AP	3.79
	Jhabua, MP	39.11	Mokokchung, Nagaland	3.11
Low birth weight	Karauli, Rajasthan	37.66	Kohima, Nagaland	3.01
	Neemuch, MP	36.99	Mamit, Mizoram	2.99
	Vidisha, MP	34.1	Lawangtlai, Mizoram	2.64

*AP, Arunachal Pradesh; J&K, Jammu & Kashmir; MP, Madhya Pradesh; UP, Uttar Pradesh*.

## 4. Discussion

Policymakers and public health specialists have been working for decades to fight against malnutrition, particularly in LMICs. The study presents four substantial findings relevant for designing interventions to alleviate nutritional status in India and perhaps other LMIC. It has been observed that the birth weight of the child, BMI of the mother, place of residence, and education of the mother had a significant positive effect. Literature states that BMI of the mothers is more pervading in India than other factors that affect birth weight (30). Women with under-education levels and those living in poorer neighborhoods were more vulnerable to adverse birth outcomes (31, 32). These findings revealed that the sex of the child and education of the mother positively affected exclusive breastfeeding practices. Studies have shown that mothers perceived breastfeeding as a surpassing substitute, such as infant formula or solid food nurses sons longer than daughters. There are also established associations between basic of a mother education and infant feeding practices worldwide (33, 34). This study also revealed that the education of the mother and wealth status significantly affected prenatal visits. A spatial analysis (35) from India revealed that literacy plays a significant role in ANC visits while illiteracy and poverty are important risk factors for having less or no prenatal care (36). Emergency transport services also play a vital role in ANC (37, 38), such as a dedicated Janani express (free transportation under Janani Sishu Suraksha 192 Karyakaram). Findings from the path also revealed that diarrhea and exclusive breastfeeding had a significant positive effect on wasting among children. Diarrhea is a major problem in India, and if not treated on time is associated with morbidity and mortality (39, 40). Initiation of breastfeeding within 1 h of birth and subsequent exclusive breastfeeding provides the infant with antibodies (Immunoglobulin A and Immunoglobulin M) required to fight against many bacteria and viruses (41–43). We found children ever born to have a positive significance on stunting, wasting, and underweight. Studies from South Asia have evidenced that son preference/daughter aversion may affect child malnutrition (44). It is also found from the literature that having brothers heighten girl risk for acute malnutrition (45, 46).

Most of the low-performing districts for exclusive breastfeeding were found to be from Uttar Pradesh in India. Previous studies (47) have stated that improved coverage of antenatal care and counseling can improve new-born care in rural areas. Literature (48) states a lack of awareness regarding Infant Young Child Feeding (IYCF) in Uttar Pradesh. IYCF program, if implemented with accountability, can reduce the neonatal mortality rate by 20% and reduce the malnutrition infection-cycle (49, 50). It will also provide short-term and long-term health and economic and environmental advantages to children, women, and society. These findings reveal that extremely poor toilet facilities are still prevailing in Bihar, Jharkhand, Odisha, Chhattisgarh, and Madhya Pradesh. Improvements in sanitation would be the most effective way to tackle chronic undernutrition and stunting (51). A study from Indonesia found that child stunting rates were high even from the wealthiest quintile of households, implying that income growth alone would not solve this issue (52). Most of the districts having poor water sources were from Maharashtra. According to a study conducted in Maharashtra in 2019, water samples studied for water quality were not found suitable for drinking (53). Literatures state that safe access to water sources reduces the probability of birth malnutrition among poor households (54). Least prenatal visits were found in Arunachal Pradesh, Nagaland, and Uttar Pradesh. These could probably because of poor accessibility and primary health services in the North-eastern part of India (55). Improved ANC coverage and removing barriers of inequalities in health would gradually help reduce child malnutrition (56). Diarrhea was found to be more prevalent in Uttar Pradesh. Emphasis should control the widespread diarrheal disease by tackling the social determinants (57) and subsequent chronic malnutrition (58). Children mainly from districts of Madhya Pradesh were found to have low birth weight. It is evidenced that low birth weight significantly contributes to malnutrition (59, 60). Documentation of children born with low birth weight is vital (61) to keep track of those children to provide special care and home visits of Accredited Social Health Activist (ASHA) as per Reproductive, Maternal, New born, Child Health + Adolescent (RMNCH+A) guidelines (62).

The quality of data used from NFHS is likely to be affected by the overstretching of the numbers of questions administered. The presented study also includes the limitations of a cross-sectional design. The relations observed in path analysis cannot be taken as casual, and as mentioned, the direction could be reversed. Spatial analysis is generalized at district aggregate and not at the individual level, which would lead to ecological fallacy.

## 5. Conclusion

In conclusion, these findings attribute to the major factors vital to child malnutrition and stand in the way of designing interventions to improve the burden of malnutrition in the country. A robust direct relation was observed for diarrhea, exclusive breastfeeding, and children born with stunting, underweight, and wasting. The variables associated indirectly with the outcome variables were the education of the mother, place of residence, and desired pregnancy. The identification of hotspots through spatial analysis would help revive control strategies in the affected area according to geographical needs. It is extensively addressed that the interventions related to health and nutrition during the first 1,000 days of life are critical to arrest the upshot of growth floundering among children. A multisectoral approach and multiple measures should be applied with a particular focus on vulnerable groups to reduce the load of malnutrition in the country. Popularizing low-cost nutritious foods among the underprivileged and improving the public distribution system (PDS) under Integrated Child Development Scheme would also lower the burden on malnutrition. Government programs like POSHAN Abhiyan or the National Nutrition Mission should be strategically revitalized and strengthened.

## Data Availability Statement

The data set utilized for the study is available with the first and corresponding authors. It can be provided upon request.

## Author Contributions

MS, BT, and PM were responsible for conception and design of the study. MA and HN performed the statistical analysis. HN and MA created plots and figures formally. BT and MS wrote the manuscript. PM and YM did a critical review. All authors have read and approved the manuscript.

## Conflict of Interest

The authors declare that the research was conducted in the absence of any commercial or financial relationships that could be construed as a potential conflict of interest.

## Publisher's Note

All claims expressed in this article are solely those of the authors and do not necessarily represent those of their affiliated organizations, or those of the publisher, the editors and the reviewers. Any product that may be evaluated in this article, or claim that may be made by its manufacturer, is not guaranteed or endorsed by the publisher.
